# Emergence of Enteric Viruses in Production Chickens Is a Concern for Avian Health

**DOI:** 10.1155/2014/450423

**Published:** 2014-01-22

**Authors:** Elena Mettifogo, Luis F. N. Nuñez, Jorge L. Chacón, Silvana H. Santander Parra, Claudete S. Astolfi-Ferreira, José A. Jerez, Richard C. Jones, Antonio J. Piantino Ferreira

**Affiliations:** ^1^Department of Pathology, School of Veterinary Medicine, University of São Paulo, Avendia Professor Orlando Marques de Paiva 87, CEP 05508-900, São Paulo, SP, Brazil; ^2^Department of Preventive Veterinary Medicine and Animal Health, School of Veterinary Medicine, University of São Paulo, Avendia Professor Orlando Marques de Paiva 87, CEP 05508-900, São Paulo, SP, Brazil; ^3^Department of Infection Biology, Institute of Global Health, University of Liverpool, Liverpool CH64 7TE, UK

## Abstract

Several viruses have been identified in recent years in the intestinal contents of chickens and turkeys with enteric problems, which have been observed in commercial farms worldwide, including Brazil. Molecular detection of these viruses in Brazil can transform to a big threat for poultry production due to risk for intestinal integrity. This disease is characterized by severely delayed growth, low uniformity, lethargy, watery diarrhea, delayed feed consumption, and a decreased conversion rate. Chicken astrovirus (CAstV), rotavirus, reovirus, chicken parvovirus (ChPV), fowl adenovirus of subgroup I (FAdV-1), and avian nephritis virus (ANV) were investigated using the conventional polymerase chain reaction (PCR) and the reverse transcription polymerase chain reaction (RT-PCR). In addition, the infectious bronchitis virus (IBV), which may play a role in enteric disease, was included. The viruses most frequently detected, either alone or in concomitance with other viruses, were IBV, ANV, rotavirus, and CAstV followed by parvovirus, reovirus, and adenovirus. This study demonstrates the diversity of viruses in Brazilian chicken flocks presenting enteric problems characterized by diarrhea, growth retard, loss weight, and mortality, which reflects the multicausal etiology of this disease.

## 1. Introduction

The economic impact of enteric virus infections on the poultry industry has been evaluated and ranges from insignificant economic effects to those that are severe and cause devastating losses. Enteric diseases tend to predominantly affect young birds; however, the disease may occur in all age groups, which increases susceptibility to other diseases, decreases feed conversion efficiency, and prolongs the time to market [1, 2]. At present, no specific treatment exists, and commercially available vaccines have not yet been developed for any of the viruses that are involved in this disease.

Enteric disease was induced by experimental infection in one-day-old broiler chicks with intestinal content from a broiler flock which has presented enteric problems such as diarrhea, poor performance, and mortality [3, 4] or with preparations from the intestinal contents of affected birds that did not contain bacteria or protozoa [3, 5]. However, experimental attempts to reproduce this disease following inoculation with a single pathogen were unsuccessful. Under field conditions, these intestinal infections are usually complicated by interactions with other infectious agents or by the age, nutrition, and immune status of the birds as well as the management and environmental conditions, which complicated the evaluation of the role of these viruses in the enteric diseases manifestation [6–8]. Enteric diseases related to viruses were firstly reported in the late 1970s and is characterized by growth deficiency, retarded feather development, diarrhea, and other abnormalities [9–11]. Although several clinical cases of enteric disease have been observed in several regions of Brazil, there is no extensive research on this syndrome, except for studies on the detection of some atypical rotaviruses in broiler chickens with enteritis [12, 13] rotavirus, reovirus, and picobirnavirus using the polyacrylamide gel electrophoresis (PAGE) technique [14].

In the past, enteric disease has been called the pale bird syndrome and helicopter wing disease and was characterized by poor growth and retarded feather development. These symptoms are observed consistently along with the other less frequent clinical signs including diarrhea, increased mortality, and pancreatic and lymphoid atrophy [6, 15]. Enteric diseases seem to be the most acceptable name for this clinical manifestation because it most appropriately reflects the consistency of the clinical findings and indicates that these cases are probably caused by the same infectious agents.

In this study, we screened seven related viruses as potential agents of enteric disease in chickens to investigate the highest number of agents and their emergence in the Brazilian poultry production.

## 2. Materials and Methods

### 2.1. Field Samples

Two hundred and eighty (280) intestinal contents samples were collected from commercial chicken from nine Brazilian states, as follows: Rio Grande do Sul, Minas Gerais, São Paulo, Paraná, Pará, Rio de Janeiro, Santa Catarina, Goiás, and Ceará. The samples were collected between 2008 and 2010. Two hundred and twelve of the samples were collected from chickens with clinical signs of enteric problems that were described as diarrhea, poor weight gain, malabsorption syndrome, culling, and mortality, and 68 samples were collected from chickens without clinical histories of enteric problems in the last three reared previously flocks. Each sample was composed of a pool of intestinal contents obtained from five chickens of whole intestine, from duodenum to the end of ileum. The age of the chickens varied from three days old to 106 weeks old broilers, broiler breeders, pullet, and layer hen flocks. Samples were preserved at −20°C until shipment to the Laboratory of Avian Diseases (Sao Paulo, SP, Brazil) and were kept at −20°C until the processing.

### 2.2. Preparation of Intestinal-Content Samples

A 1 : 5 suspension was prepared with Tris-Calcium buffer (Tris/HCl 0.1 M, CaCl_2_ 1.5 mM, pH 7.3) [16]. After 30 minutes at room temperature, under periodic homogenizations, the cell debris was removed by low-speed centrifugation (3.300 ×g for 15 min). Supernatants were stored at −20°C until analysis. DNA and RNA were extracted from supernatants using TRIzol (Invitrogen, Valencia, CA, USA) according to the manufacturer's instructions.

### 2.3. Reference Viruses

ChPV, adenovirus, CAstV, and ANV strains that had been isolated from Brazilian flocks and whose identity had been confirmed by sequencing were used as positive controls in the molecular assays. Additionally, a Massachusetts vaccine strain (H120), Nebraska calf diarrhea virus, and S1133 strains were used as controls for IBV, rotavirus, and reovirus, respectively.

### 2.4. Primers

Previously published primer sets were utilized for the reverse-transcriptase polymerase chain reaction (RT-PCR) and for PCR to detect the seven viruses screened in this study. The sequences and references are showed in Table 1.

### 2.5. DNA Virus Detection

Conventional PCR was conducted to detect chicken parvovirus and fowl adenovirus of subgroup 1, DNA, as previously described [17, 18]. Briefly, extracted DNA (2.5 *μ*L) was used as a template for PCR in a 25 *μ*L reaction mix that contained 10 pmol of each of the forward and reverse primers (as described in Table 1), 5 *μ*L of 5× buffer, 0.2 mM deoxynucleoside triphosphate (dNTP) mix, 1.25 mM MgCl_2_, and 1 U of Platinum DNA Polymerase (Invitrogen). The negative control included sterile water. Amplifications were performed in a Biometra DNA thermocycler (Biometra GmbH, Goettingen, Germany). After a 5 min incubation at 95°C, 30 cycles of amplification (94°C for 30 sec, 55°C for 1 min, and 72°C for 1 min) were performed to detect chicken parvovirus and 34 cycles of amplification (94°C for 1 min, 55°C for 45 sec, and 72°C for 1 min) to detect fowl adenovirus subgroup I. The PCRs ended with a final extension step of 10 min at 72°C. The PCR products were visualized after separation by electrophoresis in an agarose gel (1.5%) using Blue Green Dye (LGC, Sao Paulo, Brazil) to stain the DNA. The size of the amplified product was estimated using the 100 base pair DNA Ladder molecular size marker (Invitrogen).

### 2.6. RNA Virus Detection

RNA isolated from field samples was *in vitro* transcribed and amplified using the One-Step RT-PCR Kit (Qiagen, Valencia, CA, USA). Primer sets that have been previously described were used to detect CAstV, reovirus, rotavirus, and ANV as described in Table 1. The 25 *μ*L reaction mix contained 1 X Qiagen One Step RT-PCR kit reaction buffer, 320 *μ*M of each dNTP, 10 pmol of each forward and reverse primer, 1 *μ*L of Qiagen RT-PCR enzyme blend, and 2.5 *μ*L of extracted RNA. To detect IBV, the primers and reaction conditions described by Cavanagh et al. [19] were used to amplify a 179 bp fragment from the 3′untranslated region. To detect ANV and CAstV the primers sets described by Day et al. [20] were used.

## 3. Results

At least one virus was detected in 226 (80.7%) of the 280 examined samples. Of the 212 samples collected from chickens with clinical signs such as diarrhea, lethargy, poor performance, poor weight gain, malabsorption, and mortality, 183 (65.4%) were positive for one or more of the screened viruses and in 43 (15.4%) samples, at least, one enteric-diseases-related virus was detected in samples from healthy chickens (Table 2). All of the investigated viruses were detected in the intestinal samples from the commercial chickens, broiler breeders, pullet, and layer hens flocks regardless of clinical signs (Table 4). The results were analyzed according to the detection of single and concomitant (Table 2) virus in samples from chickens with and without clinical symptoms (Table 2) and the absolute number of positive samples for each virus (Table 3). In both conditions of analysis described above, the viruses with the greatest frequencies in descending order were IBV, ANV, rotavirus, and CAstV. Table 2 shows the number of combinations among the viruses that were detected in the concomitant samples. The absolute numbers (Table 3) indicate that IBV was the most frequently identified virus (42.9%), followed by ANV (29.6%), CAstV (21.1%) and rotavirus (17.1%), chicken parvovirus (12.1%), FAdV-1 (9.6%), and reovirus (7.9%).

The majority of positive samples were detected in broiler chickens 193 (85.4%) during the first seven weeks of age, being more frequent at the first four weeks, following for broiler breeders with 22 (9.7%) of positive samples, and the lowest detection of enteric viruses was present in pullet and layer hens with 4.9%, both types of birds with a homogeneous distribution among the weeks (Table 4). The minimum age was three-day-old broiler without symptoms that was positive for IBV and CAstV. In the broiler breeders and pullet/layer hens, positive samples were detected in almost all age groups from the first week in the broiler breeders and from the fourth week in pullet/layer hens (Table 4). Two samples from the breeder chicks were positive for CAstV at one day old.

## 4. Discussion

Bacteria and parasites have been considered the primary etiological agents of gastroenteritis in commercial poultry. However, many viral infections have been associated with enteric diseases of chickens and turkeys, including coronavirus, reoviruses, rotaviruses, adenoviruses, enteroviruses, and the members of Family *Astroviridae* (Chicken Astrovirus-CAstV and Avian Nephritis Virus-ANV) [22–27]. Infections with the previously mentioned viruses are believed to be important in the pathogenesis of the economically important enteric disease, such as runting and stunting syndrome (RSS), which affects young chickens, mainly broiler chickens [6, 9, 28, 29] and turkeys [1]. Recently, studies have included the enterotropic strains of infectious bronchitis virus (IBV) as a possible etiological agent of enteritis in chickens [30]. IBV can grow in many cells of the gastrointestinal tract [31], and some Asian strains were described to cause lesions in the proventriculus. IBV is believed to only persist in the gastrointestinal tract of young chickens and in layers without clinical disease [32].

Different denominations or terms have been used to describe the enteric disease in poultry because the clinical signs are infrequent or occur independently of previous conditions, such as the presence of primary or secondary etiological agents, the immune and nutritional status of the host, and environmental conditions [3, 4, 15, 33]. According to Saif [2], the gastrointestinal tract (GIT) is the primary organ of the body that is exposed and a variety of injuries against it could result in inefficient utilization of nutrients during the early stages of development. Of the various signs described for enteric disease, diarrhea and lack of normal development are the most consistently reported symptoms.

The results obtained in this study demonstrate a high level of infection with one or more of the seven viruses investigated in the chickens with clinical symptoms (65.4%), as shown in Table 2. However, samples taken from chickens without symptoms (15.4%) were also positive for these viruses (Table 2), which demonstrates a similar prevalence between these two groups. This result indicates that chickens should be shedding the virus via the enteric tract without showing any clinical symptoms; therefore, these chickens are considered asymptomatic carriers or reservoir, representing a potential source of infection. Other studies showed lower levels of rotavirus infection (4.1%) in normal chickens [14], while higher frequencies were found in asymptomatic flocks with 30% rotavirus [13] and 30% for CAstV from healthy flocks of turkeys [34]. However, these studies did not survey a wide range of viruses. In an extensive survey of turkey flocks with enteric disease and healthy turkey flocks in the United States, Rotaviruses were detected slightly more frequently in healthy than in diseased flocks [34]. Astroviruses were detected in the intestinal contents of poultry prior to the onset of clinical disease and gross pathologic changes [35]. ANV-induced clinical disease presents as kidney lesions in young chickens but only presents as a subclinical persistent infection in mature chicken [23]. These conditions reflect those viruses, other than reovirus, coronavirus, and chicken parvovirus, which have been identified in samples from flocks that appear healthy and may have different degrees of pathogenicity [22, 25]. In fact, there are indications that different serotypes and even strains within the same serotype can vary in their ability to produce illness and death [15]. Moreover, several factors influence the susceptibility of chickens to enteroviruses, such as age, passive immunity level, simultaneous infection with other pathogens, and management failures, which cause stress [14, 36, 37].

Regardless of the symptoms, most of the samples (226 samples) representing 80.7% were positive for one or more of the viruses, which demonstrates the high prevalence of these viruses in Brazilian chicken flocks (Table 2). In almost half of these positive samples (115 samples or 41.1% of total), two or more viruses were detected simultaneously, which highlights the hypothesis of multicausal etiology for the enteric disease. The distribution of the seven viruses in these concomitance samples was demonstrated and is shown in Table 2.

The combinations were scattered proportionally according to the prevalence of each virus and did not present a pattern or a constant frequency. None particular combination of the viruses was observed with significant repeatability. Rarely, a single agent is the sole contributing factor to enteric disease; moreover, the presence of different combinations of viruses could result in varied disease presentations [2].

IBV was the most prevalent virus detected in the samples that contained a single virus (Table 2) and in all of the samples (120 samples/42.9%), as shown in Table 3. IBV is the infectious agent associated with infectious bronchitis of chickens and is responsible for outbreaks in many countries, including Brazil. Infectious bronchitis (IB) is characterized by respiratory, reproductive, and, sometimes, renal signs. However, some strains of this virus have been demonstrated to multiply in intestinal cells and were referred to as possible agents of diarrhea or may at least have some role in enteric diseases [31]. Recently, we isolated variant strains of IBV from the intestinal contents of chickens with enteritis but without the typical respiratory, reproductive, or renal signs [30]. In these cases, IBV was the only pathogen present, while all of the samples were negative for astrovirus, reovirus, and rotavirus. When one-day-old SPF chicks were inoculated with filtrated IBV variants, respiratory signs but not diarrheal or renal signs were observed (data not published). In addition, these samples were not screened at for other likely agents of enteritis, such as ANV or chicken parvovirus. However, the IBV strains that were detected in the intestinal contents may not play a role as direct pathogens in enteric disease.

Of the viruses investigated, ANV was the second most prevalent by absolute numbers and was detected in 83 samples (29.6%), as shown in Table 3. If IBV is not the primary causal agent, then ANV is the most prevalent pathogen causing enteric disease that was detected in this study. Originally regarded as a picornavirus, ANV was recently characterized as a new member of the Family *Astroviridae* in 2000 [23] and has been detected in kidney samples from young chickens with growth deficiencies in Hungary [38]. A recent study that was performed in the United States to detect the presence of several enteroviruses in chicken flocks demonstrated that ANV was the most prevalent virus followed by coronavirus, reovirus, CAstV, and rotavirus [21]. The characteristic signs of avian nephritis that are caused by AVN vary from none (subclinical) to outbreaks characterized by diarrhea, growth retard, renal failure with tubulonephrosis, interstitial nephritis, uricosis, and death [37, 39].

Chicken astroviruses (CAstV) were the third and rotaviruses were the fourth most frequent agents detected in this study, with 21.1% and 17.1%, respectively (Table 3). Previously, a high frequency of CAstV was reported by Pantin-Jackwood et al. [21], where 21 of the 34 (61.7%) analyzed samples were identified as positive for CAstV. Astroviruses cause or have been associated with acute gastroenteritis in humans, cattle, swine, sheep, cats, dogs, deer, mice, and turkeys, as well as with fatal hepatitis in ducks [26, 29, 40, 41]. However, the clinical importance of CAstV remains unclear. On the other hand, rotaviruses are frequently associated with enteric disease, but the economic significance of rotaviruses to the poultry industry has not yet been defined [10, 36, 42]. Rotaviruses were present in 46.5% of the samples and in chicken flocks from all regions of the United States that were tested during 2005 and 2006 [21]. Studies on the classification of serogroups by PAGE have indicated that group D rotaviruses are the most frequently reported group in United Kingdom flocks [42]. Furthermore, group D rotavirus infection has recently been implicated as a contributing factor to the development of RSS in 5- to 14-day-old broilers in Germany [43]. The A, F, and G groups have also been detected in broiler flocks [36, 43]. In Brazil, a study identified nine distinct electropherogroups using PAGE, but only three were similar to the A group profile of avian rotavirus [14]. More recently, rotavirus was also detected in 45.3% of chicken and layer flocks in Brazil, and approximately 15% of these samples were identified as belonging to group A [13]. In a previous study using the same RT-PCR test for the NSP4 gene [22], four different genotypes of rotaviruses were detected in samples from commercial turkeys, reflecting the great genetic variability of rotaviruses similar to that reported in humans and other mammals.

Parvoviruses are known to cause gastrointestinal disease in mammalian species and have been implicated as a cause of malabsorption syndrome in chickens and enteritis in turkeys [9, 44]. However, the role of chicken parvoviruses in disease has not yet been determined. Previous studies using electron microscopy have identified parvovirus-like particles in the samples of chickens with enteric disease [45], but it was not possible to confirm the presence of parvoviruses until the development of new molecular tools such as PCR [46]. In this survey, 12.1% of the chickens were positive for chicken parvovirus, which is slightly more than those which were positive for reovirus and adenovirus and indicates that chicken parvovirus should be considered as an important etiological agent of enteric disease in chicken. In a nationwide survey in the United States, a high prevalence of chicken parvovirus (77%) was detected in 54 chicken samples [18].

Fowl Adenovirus subgroup I had a frequency 9.6% positive samples among the viruses investigated in this study (Table 3). The subgroup I and II adenoviruses are considered widely distributed in poultry and are commonly found in enteric samples [10, 47]. Although the association between adenovirus and disease is well established for subgroup II (turkey hemorrhagic enteritis and related viruses and subgroup III (egg drop syndrome)), the role of most subgroup I avian adenoviruses as pathogens is not well defined [10].

Avian reoviruses are frequently detected in the intestinal tracts of poultry with enteric disease and are widely distributed in poultry [48]. Avian reoviruses were identified in this study of lowest frequency with only 7.9% (Table 3). Avian reoviruses were identified in 62.8% of the chicken flocks tested from all regions of the United States during 2005 and 2006 [21]. Avian reoviruses have been isolated from a variety of tissues in chickens that are affected by assorted disease conditions including viral arthritis/tenosynovitis, respiratory disease, immunosuppression, and enteric disease or malabsorption syndrome [48]. Moreover, avian reoviruses have also been demonstrated to cause a synergistic effect and enhance the pathogenicity of other agents, such as chicken anemia virus, *Escherichia coli* [49], and infectious bursal disease virus [50]. However, the severity of the effects depended on the strain of reovirus. A clear relationship is frequently reported between arthritis/tenosynovitis and reovirus infection. However, the role of avian reoviruses in enteric disease remains unclear, when we consider other primary pathogens associated with enteric problems in chickens [49, 50].

The association between viruses and age showed that the viruses could be detected at different stages in the broilers, breeders, and pullet/layer hens; for example, virus was detected from the first week of age to the last week, especially in broilers. An important finding was the detection of CAstV in one-day-old breeder chicks, which may constitute an indicator of vertical transmission. According to McNulty et al. [42], in a longitudinal study carried out in young poultry flocks, the avian rotavirus is normally detected after two weeks of age because of the modulation of the passive maternal immunity. However, Tamehiro et al. [14] reported samples that were positive for rotavirus in one-week-old broiler chickens. Furthermore, Pantin-Jackwood et al. [21] demonstrated the presence of rotavirus in samples collected from poults before placement, in addition to the presence of astrovirus throughout their lifetime.

In conclusion, the primary viruses detected in this study were the IBV, ANV, CAstV, and rotavirus, followed by chicken parvovirus, adenovirus, and reovirus. However, the astroviruses (ANV and CAstV) should be regarded as the most important pathogens because coronavirus (IBV), although present in a higher percentage of samples, has not been demonstrated to cause pathogenicity in the enteric tract in our previous experiments (data not published). Detection of virus was high among chickens with and without clinical signs of disease, which demonstrates the high prevalence of these viruses in asymptomatic carriers and indicates that these carries may represent a potential source of infection. Moreover, the presence of CAstV in one-day-old breeder chicks that was detected in this study may suggest a vertical transmission. In addition, the injuries that these viruses cause can interfere with the absorption capacity of the intestinal tract during the first weeks of life of these birds, which enhances the negative impact on productivity for the rest of the production cycle. This is the first study to demonstrate the presence of these viruses in association with enteric disease in Brazil. Experimental challenges should be conducted to establish the role of these viruses and to analyze their impact on commercial chicken productivity.

## Figures and Tables

**Table 1:** Primers sets, nucleotide sequences, amplicons, and the corresponding references that were used to screen for the viruses.

Virus	Primer	Nucleotide sequence (5′-3′)	Amplicon (bp)	Reference
Parvovirus	PVF 1	GGCCGTTAACGATATCACTCAAGTTTC	561	[18]
	PVR 1	AAAGCGCTTGCGGTGAAGTCTGGCGCT		
Avian adenovirus group I	Hexon A	CAA RTT CAG RCA GAC GT	897	[17]
	Hexon B	TAG TGA TGM CGS GAC ATC AT		
IBV	UTR 41+	ATGTCTATCGCCAGGGAAATGTC	179	[6]
	UTR 31	GGGCGTCCAAGTGCTGTACCC		
	UTR 11	GCTCTAACTCTATACTAGCCTA		
CAstV	CASpolIF	GAY^A^CAR^B^CGAATGCGR^B^AGR^B^TTG	362	[20]
	CASpolIR	TCAGTGGAAGTGGGK^C^AR^B^TCTAC		
Reovirus	S4-F13	GTGCGTGTTGGAGTTTCCCG	1120	[21]
	S4-R1133	TACGCCATCCTAGCTGGA		
Rotavirus	F30	GTGCGGAAAGATGGAGAAC	630	[22]
	R660	GTTGGGGTACCAGGGATTAA		
ANV	Pol 1F	GY^A^TGGGCGCY^A^TCY^A^TTTGAY^A^AC	473	[20]
	Pol 1R	CR^B^TTTGCCCK^C^R^B^TAR^B^TCTTTR^B^T		

^A^Y: pyrimidine;^B^R: purine;^C^K: G ou T.

**Table 2:** Distribution of the seven viruses in the 280 samples of intestinal contents from the chickens with and without clinical signs of enteric diseases from 2007 to 2010.

Virus	With clinical signs %	Without clinical signs %	Total (%)
IBV	39 (13.9)	6 (2.1)	45 (16.1)
ANV	18 (6.4)	4 (1.4)	22 (7.9)
CAstV	10 (3.6)	4 (1.4)	14 (5.0)
Parvovirus	5 (1.8)	0 (0.0)	5 (1.8)
Adenovirus	12 (4.3)	2 (0.7)	14 (5.0)
Rotavirus	6 (2.1)	4 (1.4)	10 (3.6)
Reovirus	1 (0.4)	0 (0.0)	1 (0.4)

Total one virus	91 (32.5)	20 (7.1)	111 (39.6)

Concomitance^†^			

Two viruses	60 (21.4)	14 (5.0)	74 (26.4)
Three viruses	25 (8.9)	6 (2.1)	31 (11.1)
Four viruses	7 (2.5)	2 (0.7)	9 (3.2)
Five viruses	0 (0.0)	1 (0.4)	1 (0.4)

Total concomitance viruses	92 (32.9)	23 (8.2)	115 (41.1)

Total of positives	183 (65.4%)	43 (15.4%)	226 (80.7)
Negative	29 (10.4)	25 (8.9)	54 (19.3)

Total	212 (75.7)	68 (24.3)	280 (100)

IBV: infectious bronchitis virus; ANV: avian nephritis virus; CAstV: chicken astrovirus; ^†^two or more viruses detected simultaneously in the same sample.

**Table 3:** Frequencies of individual and multiple enteric virus infections detected in intestinal samples.

Number of combinations viruses detected	IBV	ANV	CAstV	Rotavirus	ChPV	FAdV-1	Reovirus
One virus	45	22	14	10	5	14	1
Two viruses	49	28	16	31	13	7	4
Three viruses	17	25	22	4	10	4	11
Four viruses	8	7	6	3	5	2	5
Five viruses	1	1	1	0	1	0	1

Number of positive samples	120	83	59	48	34	27	22

% of positive samples for each virus (*n* = 280)	42.9	29.6	21.1	17.1	12.1	9.6	7.9

ANV: avian nephritis virus; CAstV: chicken astrovirus; ChPV: chicken parvovirus; IBV: infectious bronchitis virus; FAdV-1: fowl adenovirus group 1.

**Table 4:** Positive samples for one or more viruses obtained from broiler chickens, breeders, and layer hens and the respective age in weeks at chicks which were detected.

Broiler chickens		Broiler breeders		Pullet/layer hens	
Weeks	Number of positive samples	Weeks	Number of positive samples	Weeks	Number of positive samples
1	27	1	3	4	1
2	32	8	1	8	1
3	31	12	1	13	2
4	33	23	1	23	1
5	20	31	1	24	1
6	33	32	2	31	1
7	17	33	2	48	1
		34	1	93	2
		35	1	106	1
		37	3		
		40	2		
		45	1		
		51	1		
		57	1		
		58	1		
					

Total	193 (85.4%)	Total	22 (9.7%)	Total	11 (4.9%)

% of samples positive for each type of bird (*n* = 226).
